# 4-Methyl-*N*-{4-[(5-methyl-1,2-oxazol-3-yl)sulfamo­yl]phen­yl}benzene­sulfonamide

**DOI:** 10.1107/S1600536812005260

**Published:** 2012-02-17

**Authors:** Muhammad Sohail, Muhammad Nadeem Asghar, M. Nawaz Tahir, Muhammad Shafique, Muhammad Ashfaq

**Affiliations:** a Forman Christian College (A Chartered University), Ferozepur Road, Lahore 54600, Pakistan; b University of Sargodha, Department of Physics, Sargodha, Pakistan; c Department of Chemistry, GC University, Lahore 54000, Pakistan; d University of Gujrat, Department of Chemistry, Gujrat, Pakistan

## Abstract

In the title compound, C_17_H_17_N_3_O_5_S_2_, the dihedral angle between the two benzene rings is 81.27 (8)° and the heterocyclic ring is oriented at 9.1 (2) and 76.01 (9)° with respect to these rings. Mol­ecules are connected *via* N—H⋯N and N—H⋯O hydrogen bonds, generating an *R*_2_^2^(8) motif, into chains running along the [001] direction. There is also an intra­molecular C—H⋯O hydrogen bond completing an *S*(6) ring motif. The polymeric chains are inter­linked through inter­molecular C—H⋯O hydrogen bonds.

## Related literature

For a related crystal structure, see: Ashfaq *et al.* (2010 ▶). For graph-set notation, see: Bernstein *et al.* (1995 ▶).
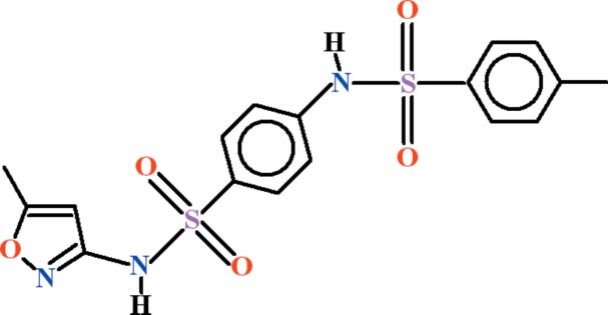


## Experimental

### 

#### Crystal data

C_17_H_17_N_3_O_5_S_2_
*M* *_r_* = 407.46
Monoclinic, 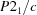

*a* = 10.6294 (6) Å
*b* = 12.2394 (7) Å
*c* = 14.9673 (11) Å
β = 106.863 (2)°
*V* = 1863.5 (2) Å^3^
*Z* = 4
Mo *K*α radiation
μ = 0.32 mm^−1^
*T* = 296 K
0.35 × 0.25 × 0.22 mm

#### Data collection

Bruker Kappa APEXII CCD diffractometer
Absorption correction: multi-scan (*SADABS*; Bruker, 2005 ▶) *T*_min_ = 0.915, *T*_max_ = 0.938
17943 measured reflections
4629 independent reflections
2488 reflections with *I* > 2σ(*I*)
*R*_int_ = 0.056

#### Refinement

*R*[*F* ^2^ > 2σ(*F* ^2^)] = 0.055
*wR*(*F* ^2^) = 0.141
*S* = 1.02
4629 reflections
246 parameters
H-atom parameters constrained
Δρ_max_ = 0.47 e Å^−3^
Δρ_min_ = −0.37 e Å^−3^

### 

Data collection: *APEX2* (Bruker, 2009 ▶); cell refinement: *SAINT* (Bruker, 2009 ▶); data reduction: *SAINT*; program(s) used to solve structure: *SHELXS97* (Sheldrick, 2008 ▶); program(s) used to refine structure: *SHELXL97* (Sheldrick, 2008 ▶); molecular graphics: *ORTEP-3 for Windows* (Farrugia, 1997 ▶) and *PLATON* (Spek, 2009 ▶); software used to prepare material for publication: *WinGX* (Farrugia, 1999 ▶) and *PLATON*.

## Supplementary Material

Crystal structure: contains datablock(s) global, I. DOI: 10.1107/S1600536812005260/gk2455sup1.cif

Structure factors: contains datablock(s) I. DOI: 10.1107/S1600536812005260/gk2455Isup2.hkl

Supplementary material file. DOI: 10.1107/S1600536812005260/gk2455Isup3.cml

Additional supplementary materials:  crystallographic information; 3D view; checkCIF report

## Figures and Tables

**Table 1:** Hydrogen-bond geometry (Å, °)

*D*—H⋯*A*	*D*—H	H⋯*A*	*D*⋯*A*	*D*—H⋯*A*
N1—H1⋯N3^i^	0.86	2.04	2.859 (3)	159
N2—H2*A*⋯O1^ii^	0.86	2.39	2.979 (3)	127
C2—H2⋯O4^iii^	0.93	2.42	3.208 (4)	143
C13—H13⋯O2	0.93	2.49	3.134 (3)	126

Symmetry codes: (i) 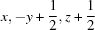; (ii) 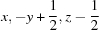; (iii) 
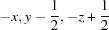
.

**Fig. 1.**
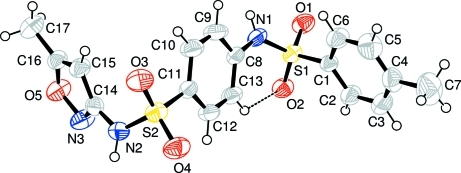
View of the title molecule with displacement ellipsoids drawn at the 50% probability level. H-atoms are shown as small circles of arbitrary radii. The dotted line represents an intramolecular C-H···O hydrogen bond

**Fig. 2.**
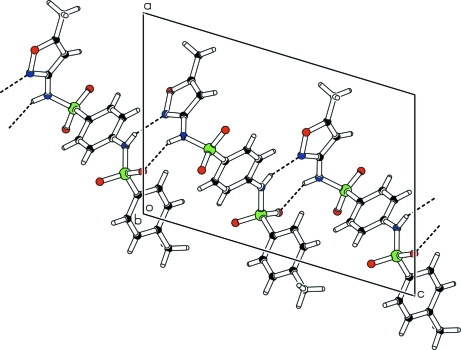
The partial packing (PLATON; Spek, 2009) which shows that molecules form polymeric chains extending along [0 0 1] direction.

## supplementary crystallographic information

### Comment

The title compound (Fig. 1) has been synthesized as a part of the series of new sulfonamide derivatives. The aim of our research work is to find the potential sulfonamide derivatives possesing anti-microbial activity. The crystal structures of a similar compound, *N*-[4-(*p*-toluenesulfonamido)phenylsulfonyl]acetamide (Ashfaq *et al.*, 2010) has already been reported.

In (I), the phenyl rings A (C1–C6), B (C8—C13) and the heterocyclic five- membered ring C (C14/C15/C16/O5/N3) are planar with r. m. s. deviation of 0.0068 Å, 0.0031 Å and 0.0058 Å, respectively. The dihedral angles between A/B, A/C and B/C are 81.27 (8)°, 9.12 (20)° and 76.01 (9)°, respectively. There exist intramolecular hydrogen bond of C—H···O type (Table 1, Fig. 1) forming an S(6) ring motif (Bernstein *et al.*, 1995). There exist intermolecular hydrogen bonds of N—H···N and N—H···O types (Table 1, Fig. 2) due to which the molecules are connected from the ends to form one-dimensional polymeric network along the [0 0 1] direction and complete *R*_2_^2^(8) ring motifs. The polymeric chains are interlinked through intermolecular hydrogen bond of C—H···O type (Table 1, Fig. 2).

### Experimental

Equimolar amount of 4-amino-*N*-(5-methyl-4,5-dihydro-1,2-oxazol-3-yl)benzenesulfonamide and *p*-toluenesulfonyl chloride was dissolved in 20 ml distilled water. The pH was adjusted to 8–9 using Na_2_CO_3_ (1 *M*) and the solution was stirred at room temperature for 6 h. The progress of reaction was monitored by the consumption of suspended *p*-toluenesulfonyl chloride. On completion, pH was adjusted to 2–3 using HCl (2 *N*). The precipitate formed was filtered, washed with distilled water and recrystallized from methanol to afford colorless prisms (m.p. 403 K).

### Refinement

The H-atoms were positioned geometrically (N–H = 0.86 Å, C–H = 0.93–0.96 Å) and refined as riding with *U*_iso_(H) = *xU*_eq_(C, N), where *x* = 1.5 for methyl groups and *x* = 1.2 for all H-atoms.

### Crystal data

**Table:**

C_17_H_17_N_3_O_5_S_2_	*F*(000) = 848
*M**_r_* = 407.46	*D*_x_ = 1.452 Mg m^−^^3^
Monoclinic, *P*2_1_/*c*	Mo *K*α radiation, λ = 0.71073 Å
Hall symbol: -P 2ybc	Cell parameters from 2488 reflections
*a* = 10.6294 (6) Å	θ = 2.2–28.3°
*b* = 12.2394 (7) Å	µ = 0.32 mm^−^^1^
*c* = 14.9673 (11) Å	*T* = 296 K
β = 106.863 (2)°	Prism, colorless
*V* = 1863.5 (2) Å^3^	0.35 × 0.25 × 0.22 mm
*Z* = 4	

### Data collection

**Table:**

Bruker Kappa APEXII CCD diffractometer	4629 independent reflections
Radiation source: fine-focus sealed tube	2488 reflections with *I* > 2σ(*I*)
Graphite monochromator	*R*_int_ = 0.056
Detector resolution: 7.50 pixels mm^-1^	θ_max_ = 28.3°, θ_min_ = 2.2°
ω scans	*h* = −14→13
Absorption correction: multi-scan (*SADABS*; Bruker, 2005)	*k* = −14→16
*T*_min_ = 0.915, *T*_max_ = 0.938	*l* = −19→19
17943 measured reflections	

### Refinement

**Table:**

Refinement on *F*^2^	Primary atom site location: structure-invariant direct methods
Least-squares matrix: full	Secondary atom site location: difference Fourier map
*R*[*F*^2^ > 2σ(*F*^2^)] = 0.055	Hydrogen site location: inferred from neighbouring sites
*wR*(*F*^2^) = 0.141	H-atom parameters constrained
*S* = 1.02	*w* = 1/[σ^2^(*F*_o_^2^) + (0.0577*P*)^2^ + 0.1435*P*] where *P* = (*F*_o_^2^ + 2*F*_c_^2^)/3
4629 reflections	(Δ/σ)_max_ < 0.001
246 parameters	Δρ_max_ = 0.47 e Å^−^^3^
0 restraints	Δρ_min_ = −0.37 e Å^−^^3^

### Special details

**Table:**

Geometry. Bond distances, angles *etc*. have been calculated using the rounded fractional coordinates. All su's are estimated from the variances of the (full) variance-covariance matrix. The cell e.s.d.'s are taken into account in the estimation of distances, angles and torsion angles
Refinement. Refinement of *F*^2^ against ALL reflections. The weighted *R*-factor *wR* and goodness of fit *S* are based on *F*^2^, conventional *R*-factors *R* are based on *F*, with *F* set to zero for negative *F*^2^. The threshold expression of *F*^2^ > σ(*F*^2^) is used only for calculating *R*-factors(gt) *etc*. and is not relevant to the choice of reflections for refinement. *R*-factors based on *F*^2^ are statistically about twice as large as those based on *F*, and *R*- factors based on ALL data will be even larger.

### Fractional atomic coordinates and isotropic or equivalent isotropic displacement parameters (Å^2^)

**Table:**

	*x*	*y*	*z*	*U*_iso_*/*U*_eq_	
S1	0.17178 (7)	0.12476 (6)	0.43330 (5)	0.0475 (3)	
S2	0.42023 (7)	0.58034 (6)	0.24478 (5)	0.0485 (3)	
O1	0.20899 (18)	0.03512 (15)	0.49755 (15)	0.0596 (8)	
O2	0.09435 (18)	0.10311 (16)	0.33965 (14)	0.0575 (7)	
O3	0.54004 (19)	0.62547 (15)	0.30218 (14)	0.0594 (7)	
O4	0.30436 (19)	0.64578 (16)	0.21489 (15)	0.0642 (8)	
O5	0.65024 (19)	0.33654 (16)	0.10010 (15)	0.0618 (8)	
N1	0.3101 (2)	0.18092 (19)	0.43406 (16)	0.0519 (8)	
N2	0.4456 (2)	0.53685 (18)	0.14776 (16)	0.0486 (8)	
N3	0.5291 (2)	0.3918 (2)	0.08307 (18)	0.0605 (9)	
C1	0.0863 (2)	0.2224 (2)	0.47933 (18)	0.0398 (8)	
C2	−0.0473 (3)	0.2308 (3)	0.4409 (2)	0.0573 (11)	
C3	−0.1174 (3)	0.3048 (3)	0.4774 (2)	0.0684 (14)	
C4	−0.0567 (3)	0.3690 (3)	0.5522 (2)	0.0615 (12)	
C5	0.0766 (4)	0.3572 (3)	0.5909 (3)	0.0703 (14)	
C6	0.1495 (3)	0.2850 (3)	0.5556 (2)	0.0592 (11)	
C7	−0.1339 (4)	0.4484 (3)	0.5932 (3)	0.1003 (19)	
C8	0.3303 (2)	0.2762 (2)	0.38676 (19)	0.0440 (9)	
C9	0.4465 (3)	0.3330 (3)	0.4242 (2)	0.0607 (11)	
C10	0.4738 (3)	0.4247 (3)	0.3811 (2)	0.0578 (11)	
C11	0.3859 (2)	0.4623 (2)	0.30027 (18)	0.0427 (9)	
C12	0.2695 (3)	0.4061 (2)	0.2627 (2)	0.0509 (10)	
C13	0.2425 (3)	0.3132 (2)	0.3054 (2)	0.0513 (10)	
C14	0.5480 (3)	0.4644 (2)	0.14888 (19)	0.0430 (9)	
C15	0.6744 (3)	0.4591 (2)	0.2104 (2)	0.0498 (10)	
C16	0.7342 (3)	0.3799 (2)	0.1768 (2)	0.0482 (10)	
C17	0.8701 (3)	0.3364 (3)	0.2031 (2)	0.0661 (11)	
H1	0.37992	0.14872	0.46739	0.0623*	
H2	−0.09040	0.18700	0.39052	0.0687*	
H2A	0.39496	0.55907	0.09507	0.0583*	
H3	−0.20773	0.31110	0.45045	0.0821*	
H5	0.11898	0.39923	0.64258	0.0842*	
H6	0.23972	0.27863	0.58283	0.0707*	
H7A	−0.22555	0.42985	0.57205	0.1503*	
H7B	−0.12163	0.52121	0.57344	0.1503*	
H7C	−0.10358	0.44443	0.66009	0.1503*	
H9	0.50638	0.30849	0.47898	0.0729*	
H10	0.55227	0.46190	0.40663	0.0696*	
H12	0.20926	0.43125	0.20833	0.0610*	
H13	0.16469	0.27516	0.27928	0.0614*	
H15	0.70953	0.50155	0.26337	0.0597*	
H17A	0.90803	0.34876	0.15310	0.0992*	
H17B	0.86837	0.25935	0.21492	0.0992*	
H17C	0.92184	0.37272	0.25840	0.0992*	

### Atomic displacement parameters (Å^2^)

**Table:**

	*U*^11^	*U*^22^	*U*^33^	*U*^12^	*U*^13^	*U*^23^
S1	0.0381 (4)	0.0495 (4)	0.0522 (5)	0.0024 (3)	0.0088 (3)	−0.0024 (4)
S2	0.0440 (4)	0.0468 (4)	0.0504 (5)	0.0060 (3)	0.0071 (3)	−0.0020 (3)
O1	0.0506 (12)	0.0490 (12)	0.0743 (15)	0.0042 (10)	0.0104 (10)	0.0135 (11)
O2	0.0497 (11)	0.0649 (13)	0.0521 (13)	0.0017 (10)	0.0054 (10)	−0.0180 (10)
O3	0.0567 (12)	0.0535 (12)	0.0586 (14)	−0.0064 (10)	0.0019 (11)	−0.0103 (10)
O4	0.0588 (13)	0.0586 (13)	0.0694 (15)	0.0221 (11)	0.0095 (11)	0.0017 (11)
O5	0.0484 (12)	0.0645 (13)	0.0666 (15)	0.0060 (11)	0.0075 (11)	−0.0149 (11)
N1	0.0332 (12)	0.0610 (15)	0.0591 (16)	0.0086 (11)	0.0097 (11)	0.0108 (13)
N2	0.0456 (13)	0.0547 (14)	0.0425 (14)	0.0071 (12)	0.0083 (11)	0.0061 (11)
N3	0.0435 (14)	0.0665 (17)	0.0629 (18)	0.0073 (13)	0.0018 (13)	−0.0123 (14)
C1	0.0350 (14)	0.0425 (15)	0.0391 (15)	−0.0024 (12)	0.0063 (12)	−0.0003 (12)
C2	0.0443 (16)	0.074 (2)	0.0498 (19)	0.0014 (16)	0.0076 (14)	−0.0161 (16)
C3	0.0527 (19)	0.087 (3)	0.065 (2)	0.0168 (18)	0.0165 (17)	−0.005 (2)
C4	0.073 (2)	0.058 (2)	0.062 (2)	0.0090 (18)	0.0330 (19)	0.0019 (17)
C5	0.082 (3)	0.063 (2)	0.065 (2)	−0.014 (2)	0.020 (2)	−0.0241 (18)
C6	0.0465 (16)	0.066 (2)	0.059 (2)	−0.0070 (16)	0.0059 (15)	−0.0122 (17)
C7	0.128 (4)	0.084 (3)	0.105 (3)	0.030 (3)	0.059 (3)	−0.007 (2)
C8	0.0335 (14)	0.0518 (17)	0.0465 (17)	0.0084 (14)	0.0113 (13)	−0.0003 (14)
C9	0.0451 (17)	0.071 (2)	0.053 (2)	−0.0002 (16)	−0.0063 (15)	0.0160 (17)
C10	0.0416 (16)	0.068 (2)	0.054 (2)	−0.0052 (16)	−0.0018 (14)	0.0076 (17)
C11	0.0353 (14)	0.0511 (16)	0.0409 (16)	0.0056 (13)	0.0098 (12)	−0.0021 (13)
C12	0.0399 (16)	0.0602 (19)	0.0451 (17)	0.0064 (15)	0.0007 (14)	0.0041 (15)
C13	0.0353 (14)	0.061 (2)	0.0507 (18)	−0.0007 (14)	0.0017 (13)	−0.0019 (16)
C14	0.0414 (16)	0.0438 (16)	0.0438 (16)	−0.0055 (14)	0.0126 (13)	0.0034 (14)
C15	0.0392 (15)	0.0574 (19)	0.0480 (17)	−0.0066 (14)	0.0052 (14)	−0.0071 (15)
C16	0.0384 (15)	0.0499 (17)	0.0537 (19)	−0.0048 (14)	0.0094 (14)	0.0042 (15)
C17	0.0455 (17)	0.067 (2)	0.083 (2)	0.0075 (16)	0.0141 (17)	−0.0025 (18)

### Geometric parameters (Å, º)

**Table:**

S1—O1	1.437 (2)	C8—C9	1.386 (4)
S1—O2	1.429 (2)	C9—C10	1.367 (5)
S1—N1	1.620 (2)	C10—C11	1.376 (4)
S1—C1	1.758 (3)	C11—C12	1.384 (4)
S2—O3	1.425 (2)	C12—C13	1.375 (4)
S2—O4	1.428 (2)	C14—C15	1.393 (4)
S2—N2	1.641 (2)	C15—C16	1.335 (4)
S2—C11	1.757 (3)	C16—C17	1.482 (5)
O5—N3	1.411 (3)	C2—H2	0.9300
O5—C16	1.342 (4)	C3—H3	0.9300
N1—C8	1.413 (3)	C5—H5	0.9300
N2—C14	1.400 (4)	C6—H6	0.9300
N3—C14	1.298 (4)	C7—H7A	0.9600
N1—H1	0.8600	C7—H7B	0.9600
N2—H2A	0.8600	C7—H7C	0.9600
C1—C2	1.373 (4)	C9—H9	0.9300
C1—C6	1.377 (4)	C10—H10	0.9300
C2—C3	1.382 (5)	C12—H12	0.9300
C3—C4	1.367 (4)	C13—H13	0.9300
C4—C7	1.512 (5)	C15—H15	0.9300
C4—C5	1.374 (5)	C17—H17A	0.9600
C5—C6	1.378 (5)	C17—H17B	0.9600
C8—C13	1.378 (4)	C17—H17C	0.9600
			
O1—S1—O2	118.90 (12)	C8—C13—C12	120.2 (3)
O1—S1—N1	104.26 (12)	N2—C14—N3	118.4 (3)
O1—S1—C1	109.27 (12)	N2—C14—C15	129.4 (2)
O2—S1—N1	110.41 (12)	N3—C14—C15	112.2 (3)
O2—S1—C1	107.11 (12)	C14—C15—C16	105.0 (3)
N1—S1—C1	106.26 (12)	O5—C16—C17	116.1 (2)
O3—S2—O4	120.68 (12)	C15—C16—C17	134.0 (3)
O3—S2—N2	108.17 (12)	O5—C16—C15	109.8 (3)
O3—S2—C11	107.80 (12)	C1—C2—H2	120.00
O4—S2—N2	104.65 (12)	C3—C2—H2	120.00
O4—S2—C11	109.32 (12)	C2—C3—H3	119.00
N2—S2—C11	105.17 (12)	C4—C3—H3	119.00
N3—O5—C16	108.1 (2)	C4—C5—H5	119.00
S1—N1—C8	128.05 (18)	C6—C5—H5	119.00
S2—N2—C14	121.44 (19)	C1—C6—H6	121.00
O5—N3—C14	105.0 (2)	C5—C6—H6	121.00
C8—N1—H1	116.00	C4—C7—H7A	109.00
S1—N1—H1	116.00	C4—C7—H7B	109.00
S2—N2—H2A	119.00	C4—C7—H7C	109.00
C14—N2—H2A	119.00	H7A—C7—H7B	109.00
S1—C1—C6	121.3 (2)	H7A—C7—H7C	109.00
C2—C1—C6	120.2 (3)	H7B—C7—H7C	110.00
S1—C1—C2	118.4 (2)	C8—C9—H9	120.00
C1—C2—C3	119.6 (3)	C10—C9—H9	120.00
C2—C3—C4	121.3 (3)	C9—C10—H10	120.00
C3—C4—C5	118.0 (3)	C11—C10—H10	120.00
C3—C4—C7	121.3 (3)	C11—C12—H12	120.00
C5—C4—C7	120.7 (3)	C13—C12—H12	120.00
C4—C5—C6	122.1 (4)	C8—C13—H13	120.00
C1—C6—C5	118.8 (3)	C12—C13—H13	120.00
N1—C8—C9	117.3 (2)	C14—C15—H15	128.00
N1—C8—C13	123.5 (2)	C16—C15—H15	128.00
C9—C8—C13	119.2 (3)	C16—C17—H17A	110.00
C8—C9—C10	120.5 (3)	C16—C17—H17B	109.00
C9—C10—C11	120.4 (3)	C16—C17—H17C	109.00
S2—C11—C12	120.0 (2)	H17A—C17—H17B	109.00
C10—C11—C12	119.3 (3)	H17A—C17—H17C	110.00
S2—C11—C10	120.6 (2)	H17B—C17—H17C	109.00
C11—C12—C13	120.3 (3)		
			
O1—S1—N1—C8	−176.4 (2)	O5—N3—C14—C15	−1.4 (3)
O2—S1—N1—C8	54.8 (3)	S1—C1—C2—C3	178.3 (2)
C1—S1—N1—C8	−61.0 (3)	C6—C1—C2—C3	2.0 (5)
O1—S1—C1—C2	−106.2 (2)	S1—C1—C6—C5	−177.5 (3)
O1—S1—C1—C6	70.1 (3)	C2—C1—C6—C5	−1.3 (5)
O2—S1—C1—C2	23.9 (3)	C1—C2—C3—C4	−1.0 (5)
O2—S1—C1—C6	−159.9 (2)	C2—C3—C4—C5	−0.6 (5)
N1—S1—C1—C2	141.9 (2)	C2—C3—C4—C7	−178.8 (3)
N1—S1—C1—C6	−41.9 (3)	C3—C4—C5—C6	1.2 (6)
O3—S2—N2—C14	53.7 (2)	C7—C4—C5—C6	179.5 (3)
O4—S2—N2—C14	−176.5 (2)	C4—C5—C6—C1	−0.3 (5)
C11—S2—N2—C14	−61.3 (2)	N1—C8—C9—C10	178.7 (3)
O3—S2—C11—C10	−4.6 (3)	C13—C8—C9—C10	0.1 (5)
O3—S2—C11—C12	175.4 (2)	N1—C8—C13—C12	−179.3 (3)
O4—S2—C11—C10	−137.5 (2)	C9—C8—C13—C12	−0.8 (4)
O4—S2—C11—C12	42.5 (3)	C8—C9—C10—C11	0.4 (5)
N2—S2—C11—C10	110.6 (2)	C9—C10—C11—S2	179.8 (2)
N2—S2—C11—C12	−69.4 (2)	C9—C10—C11—C12	−0.2 (4)
C16—O5—N3—C14	0.7 (3)	S2—C11—C12—C13	179.5 (2)
N3—O5—C16—C15	0.4 (3)	C10—C11—C12—C13	−0.5 (4)
N3—O5—C16—C17	−176.7 (2)	C11—C12—C13—C8	1.0 (4)
S1—N1—C8—C9	154.9 (2)	N2—C14—C15—C16	−176.3 (3)
S1—N1—C8—C13	−26.6 (4)	N3—C14—C15—C16	1.6 (3)
S2—N2—C14—N3	145.2 (2)	C14—C15—C16—O5	−1.1 (3)
S2—N2—C14—C15	−37.0 (4)	C14—C15—C16—C17	175.2 (3)
O5—N3—C14—N2	176.8 (2)		

### Hydrogen-bond geometry (Å, º)

**Table:**

*D*—H···*A*	*D*—H	H···*A*	*D*···*A*	*D*—H···*A*
N1—H1···N3^i^	0.86	2.04	2.859 (3)	159
N2—H2*A*···O1^ii^	0.86	2.39	2.979 (3)	127
C2—H2···O4^iii^	0.93	2.42	3.208 (4)	143
C13—H13···O2	0.93	2.49	3.134 (3)	126

Symmetry codes: (i) *x*, −*y*+1/2, *z*+1/2; (ii) *x*, −*y*+1/2, *z*−1/2; (iii) −*x*, *y*−1/2, −*z*+1/2.
